# Medicine

**Published:** 1895-03

**Authors:** Theodore Fisher


### Progress of the Medical Sciences.

### MEDICINE.

Diphtheria continues to hold the most prominent place in medical literature; but as those interested in the advance of medicine have no doubt followed the leading facts, recapitulation is not needed here.

Reference may, however, be made to one point; namely, the question whether the curative action of antitoxin is in any way counterbalanced by deleterious effects in certain cases. Numerous instances of erythema are recorded, but they are not of serious nature. Mr. Lennox Browne, however, has suggested that the antitoxin sets up nephritis, which may lead to the death of the patient by anuria. Dr. Goodall, in reply to this suggestion, has pointed out that anuria is not uncommon in cases of diphtheria untreated by antitoxin, and that this complication belongs therefore to the disease and not to the treatment[1]. He supports this answer by publishing a paper giving details of several cases[2]. Dr. Goodall in that paper states that although anuria is present, there is generally little if any nephritis. The suppression of urine is therefore difficult of explanation.

One other point, brought forward by Dr. Sydney Phillips

[1] *Brit. M. J.*, 1894, vol. ii., p. 1485. [2] *Lancet*, 1895, vol. i., p. 269.

at a recent meeting of the Clinical Society, may be mentioned.[1] Dr. Phillips stated that the liability to cardiac failure during convalescence from diphtheria must be remembered when statistics of cures from antitoxin treatment are published. It is not probable that many cases treated with antitoxin will die of cardiac failure after they have left the hospitals or have passed out of the hands of their medical attendants; but it is well to bear in mind that sudden death from this cause may occur when the patient is supposed to be returning to perfect health. Antitoxin may have the power of arresting the disease, but it cannot repair any organic change that may have taken place in the heart-muscle before the treatment is commenced. This fact is a plea for the early employment of antitoxin. The common cause of death in diphtheria, whether it occurs early or late, is cardiac failure; and to preserve the heart from the poisons of diphtheria, the treatment ought to be commenced in the first forty-eight hours.

Since, however, antitoxin may not always be at hand, a preparation suggested by Loeffler, which comes before the medical world with the weight of his authority, is worthy of consideration. As is well known, the diphtheria bacilli multiply superficially in the membrane and do not enter the blood, poisons probably of the nature of ferments being absorbed. It follows that if the bacilli could be destroyed *in situ*, the entrance of fresh poisons would be at once arrested. At such a result Loeffler aims, and while not wishing to put his method before treatment by antitoxins, considers that it may be a useful adjunct. The preparation is as follows: Toluol ℥j., alcohol ℥ij., liq. ferri perchlor. ʒj.; to which is added 10 per cent. of menthol, to relieve the pain of application. It is applied every three hours.[2]

Another question that has exercised the medical mind has been the possibility of infection with typhoid fever by eating oysters. In the *British Medical Journal* of January 12th, Sir William Broadbent gave brief notes of several cases in which he considered that infection was derived from this source; and in the *Journal* of the same week some exceedingly interesting, and one would think conclusive, facts are referred to in a leading article. It is not necessary to repeat them in full here, but briefly stated the following are the main points: Several students of a university in Connecticut, who had combined themselves into fraternities, were attacked with typhoid fever. Only the members of the fraternities suffered, and it was discovered that they had partaken of supper in which oysters formed one of the articles of diet. The matter was traced further, and it was found that the oysters had been deposited for a few days in a river close to the mouth of a private sewer.

[1] *Brit. M. J.*, 1895, vol. i., p. 252. [2] *Med. Week*, vol. ii., 1894, p. 449.

This sewer was connected with a house in which a lady and her daughter had recently suffered from typhoid fever.

Dr. Percy Kidd[1] records a case in which paracentesis of the pericardium appeared to act beneficially. After the second aspiration, when twenty-eight ounces of fluid were removed, the good effect of relieving pressure upon the heart was shown by increase in the amount of urine and by the dyspnœa being less marked. The case was brought before the Medical Society of London, where Dr. Sansom and other speakers[2] showed that they were not very favourable to the operation. Dr. Sansom drew attention to the fact that it must be remembered that death in pericarditis with effusion is not, in all probability, brought about by the pressure of fluid upon the heart. The effusion is merely the evidence of a serious heart-affection that kills only too often without the presence of any fluid. While Dr. Sansom and others seemed to doubt the value of paracentesis in the effusion of acute or sub-acute pericarditis, there is one condition to which they did not refer upon which we should have liked to have heard an expression of opinion. A large chronic effusion may sometimes be met with in the pericardial cavity: an effusion so extensive that it would be impossible to mistake it for enlargement of the heart, while careful marking-out of the area of dulness would distinguish it from a pleural effusion. Unfortunately such an effusion is most common in the later stages of chronic kidney disease, when permanent recovery or even prolonged improvement is almost hopeless; yet the danger of aspiration must be so small and the probability of giving relief so great, that one would think it cannot but be advisable.

More interesting than the case of Dr. Percy Kidd is one published by Dr. A. T. Sloan.[3] The heart, instead of the pericardial cavity, was accidentally tapped, to which accident the patient apparently owed her life. A young lady, aged 19, was attacked with pericarditis while suffering from rheumatism. She became seriously ill, and Dr. Byrom Bramwell was called in consultation. Effusion was thought to be present, and as her condition gradually grew worse Dr. Sloan wished to aspirate. The friends, however, objected and desired the opinion of Dr. Bramwell. While Dr. Bramwell was being sent for, the respiration and pulse suddenly ceased. Dr. Sloan somewhat excitedly plunged the aspirating needle in the 4th left interspace, close to the sternum; but instead of fluid, eight to ten ounces of blood flowed into the bottle. While Dr. Sloan was naturally thinking that all was over he felt a movement of the heart, then a sudden jump, after which it restarted in "the race for life." The pulse became running and uncountable, and the patient delirious and finally maniacal. Dr. Bramwell

[1] *Lancet*, 1895, vol. i., p. 275. [2] *Brit. M. J.*, 1895, vol. i., p. 252.
[3] *Edinb. M. J.*, vol. xl., pt. ii., 1895, p. 673.

arrived. Ether and morphia were injected. The mania became less marked, and in an hour and a half she recovered sufficiently to recognise her mother. From that time the patient slowly improved and regained perfect health. Other cases are referred to by Dr. Sloan in which recovery took place after heart-puncture; and in conclusion he states his belief that in some cases of cardiac failure, such as occur in pneumonia, bronchitis, and some forms of heart disease, abstraction of blood from the right ventricle by means of a morphia syringe might be beneficial.

The heart is a puzzling organ, and in some instances unexpectedly recovers strength when it appears to be on the point of ceasing to beat for ever. As an example, I may mention a case that occurred in Guy's Hospital when I was house physician. A woman, aged about 45, had the orthopnœa, cyanosis, and œdema of legs of chronic cardiac disease. On three occasions about fifty ounces of fluid had been withdrawn from the chest. In the middle of one night I was called to see her. On arriving in the ward, she appeared to be dead; the nurse, an experienced one, said that she was dead. The heart, however, had not quite ceased to beat, and taking out my morphia syringe, I injected from ten to twelve syringefuls of brandy. In the course of half-a-minute to a minute there were signs of renewed cardiac action. From that time the patient steadily improved, and left the hospital. She not only lost the symptoms of cardiac disease, but recovered her energy, and when about the ward was the most ready of the patients to render help to the nurses.

Such cases reveal our want of knowledge of the heart's powers. Experience leads us to believe that all forms of stimulation, whether mechanical or medicinal, will fail to revive a heart that appears to be at the end of a downward career; yet occasionally some development that we consider remarkable disturbs our preconceived notions. In all probability we have very much to learn as regards treatment. Dr. Sloan drew blood from an overloaded heart, and the patient apparently dead recovered. Dr. Leonard Hill[1] shows, on the other hand, that the heart may stop, not from over-distension, but from want of blood. The greater part of the blood of the body may be collected in the splanchnic area, and mechanical expression will possibly restart a failing heart. In the future it may be that we shall have to study the condition of the arteries and veins as carefully as the heart itself, and having studied them, find methods, possibly in the form of external stimuli, to modify their calibre and tension. Attention should be given to Dr. George Oliver's observations[2] upon the variations in the size of the radial artery.

[1] *Lancet*, 1895, vol. i., p. 338. [2] *Pulse-Gauging*. 1895.

Dr. Ainslie Hollis writes upon the pathology of atheroma. He considers that small-cell infiltration always precedes the degenerative changes, and that the disease is probably microbic in origin.[1] Dr. Hollis has been preceded by Lancereaux in attributing at least one form of atheroma of the aorta to infection from without.[2] Lancereaux speaks of malaria as a cause. The form to which Lancereaux refers is most commonly situated just above the aortic valves, but may occur in well-defined raised patches over a considerable area. This form, which is generally attributed to syphilis, shows small-cell infiltration of the vessel-wall in the early stages. On reading Dr. Hollis's paper, one cannot help thinking that he has failed to differentiate this form from the variety that mainly affects the internal coat. Dr. Hollis found a history of syphilis in only three of fifty-two cases, yet a glance through these cases shows that several are of the nature that Lancereaux attributes to malaria, and that others would consider due to syphilis. On the other hand, it may be remarked that of nine cases noticed in the Guy's Hospital records in which the disease was well marked and of this localised character, five gave *post-mortem* evidence of syphilis or had a history of venereal disease, while only two had been abroad. In one case of severe atheroma just above the valves that recently occurred in the Bristol General Hospital there was also a history of gonorrhœa, which always suggests the possibility of syphilis. The pathology of this form of disease of aorta is of some importance, since its consequences, such as aneurism, aortic regurgitation, and angina pectoris, are so serious. Lancereaux writes on the last-mentioned manifestation—namely, angina pectoris.[3] He considers that the most severe forms of cardiac pain are due to disease of the aorta just above the aortic valves, and says that the nerves of the cardiac plexus running in the sheath of the vessel become affected. In connection with this point, it may be interesting to state that I have recently cut sections of the aorta affected with this form of aortitis. Many of the nerves in the sheath were surrounded by leucocytes. To return, however, to the remarks of Lancereaux, it is satisfactory to learn that, although he attributes the disease of the aorta that gives rise to the pain to malaria, he considers that iodide of potassium is the best remedy. It follows, then, that whether we consider the disease to be due to syphilis or not, it may be well to bear in mind that when a comparatively young or middle-aged man comes under observation complaining of cardiac pain, and on auscultation a high-pitched or somewhat clanging aortic second sound is heard suggesting some dilatation of the aorta just above the valves, iodide of potassium may possibly prove beneficial. In connection with this use of iodide of potassium,

[1] *J. Path. & Bacteriol.*, vol. iii., 1894, p. 1. [2] *Bull. méd.*, 1893. Quoted in *Annual Univ. Med. Sci.*, 1894, vol. i., B—39.

[3] *Arch. gén. de méd.*, Aôut, 1894.

it is noteworthy that Professor Fraser, of Edinburgh, lays stress upon the value of the drug in cases of aortic valve disease. Aortic valve disease and atheroma of the aorta in the neighbourhood of the valves are often intimately associated, and clinically it may be impossible to say whether the lesion is primarily one of the artery or of the valves. Lancereaux finds iodide of potassium useful in cardiac pain, and Professor Fraser in aortic regurgitation. Possibly they have both been treating manifestations of the same disease.[1]

THEODORE FISHER.

